# Correspondence

**Published:** 1872-06

**Authors:** E. T. Henry

**Affiliations:** Vicksburg, Miss.


### CORRESPONDENCE.

---

VICKSBURG, MISS., April 29th, 1872.

*Editors Companion:*

I have the pleasure to report the following case, upon the statement of Dr. D. B. Nailer, one of the best physicians of our country.

Dr. Nailer was called, recently, to see Mrs. J——, a strong, hearty woman, who was in labor with her third child. The presentation was natural in first position, and everything proceeded satisfactory, until the head was born. The child cried lustily.

Examination with the finger, demonstrated the cord was not around the neck. As the next pain came on slowly, the Dr. tried to pass the finger into the axilla to bring down the shoulder, in which he was not successful, when a very severe pain came on, but instead of drawing out the body, as is customary on such occasions, it was retroacting, drawing the head back forcibly into the vulva. So great was the "back" action, that the Dr. is of the opinion that had the chin been flexed upon the chest, the head would have been carried back into the vagina. The uterus was so firmly contracted around the neck of the child, that Dr. Nailer could not pass his finger within the os. This condition of affairs lasted half an hour. When expulsive pains came on, and the child was expelled without trouble—but dead.

Dr. W. thinks the child must have been killed by the contraction of the os uteri around the *neck* of the child. The neck was not dislocated, as was much feared. The Dr. not having any remedies with him, had not given any to assist labor, and could not give any to relieve the perverted action of the womb.

In spite of Marshall Hall's ready method, and other appliances, the child could not be resuscitated.

The placenta followed the child in quick succession, the uterus contracting very firmly and remaining so. There was no farther difficulty.

Respectfully submitted, by your ob't ser'vt,

E. T. HENRY.

